# Supplementary material for: Maternal High-Fat Diet Promotes Abdominal Aortic Aneurysm Expansion in Adult Offspring by Epigenetic Regulation of IRF8-Mediated Osteoclast-like Macrophage Differentiation
Source: Cells. 2021 Aug 27;10(9):2224. doi: 10.3390/cells10092224 (PMC8466477; doi:10.3390/cells10092224)
Supplement: Supplementary file 1 [file cells-10-02224-s001.zip › cells-1352429-supplementaryú¿1ú⌐/cells-1352429-supplementary.pdf]

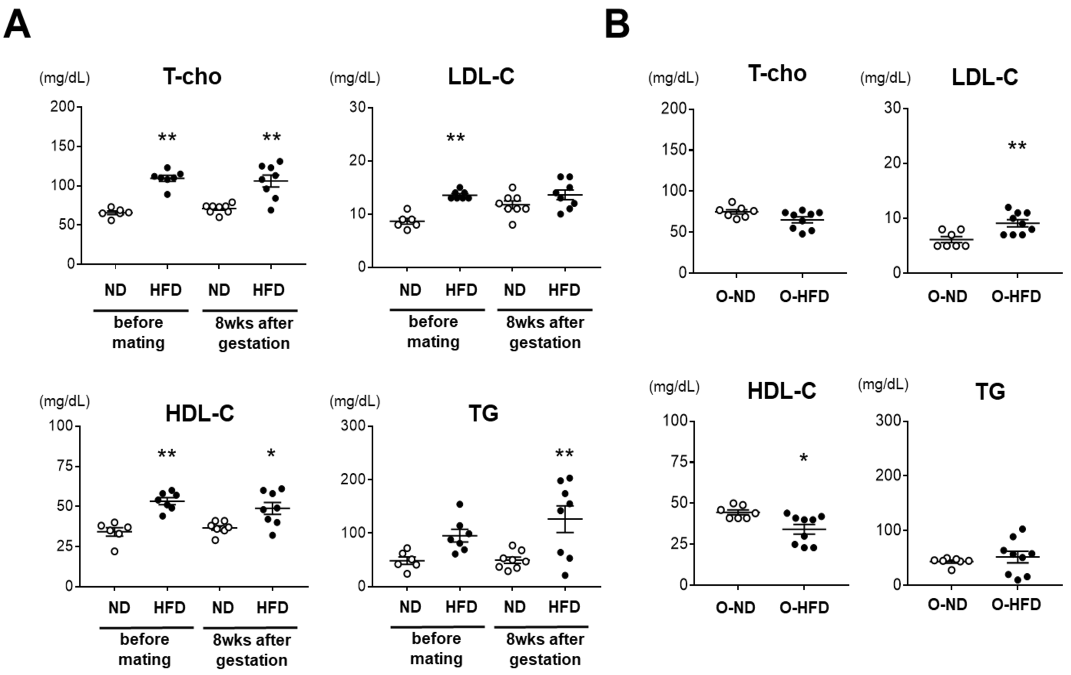

Fig. S1. Lipid profiles in dams and offspring. (A) Values are mean ± SE for 6 ND-fed dams and 7 HFD-fed dams before mating, and for 8 ND-fed dams and 8 HFD-fed dams 8 weeks after gestation. *$p < 0.05$ vs. ND-fed dams before mating. Statistical analysis made by Student t test or 2-way ANOVA with Tukey-Kramer *post hoc* test except for LDL-C and TG of dams (Kruskal-Wallis test) and LDL-C of offspring (Mann–Whitney U test). T-chol, Total cholesterol; LDL-C, Low-density lipoprotein cholesterol; HDL-C, High-density lipoprotein cholesterol; TG, Triglyceride. **(B)** Values are mean ± SE for 7 O-ND and 9 O-HFD at 8 weeks of age. *$p < 0.05$ vs. O-ND. Statistical analysis made by one-way ANOVA with Tukey-Kramer post hoc test. O-ND, offspring of ND-fed dam; O-HFD, offspring of HFD-fed dam. T-chol, Total cholesterol; LDL-C, Low-density lipoprotein cholesterol; HDL-C, High-density lipoprotein cholesterol; TG, Triglyceride.

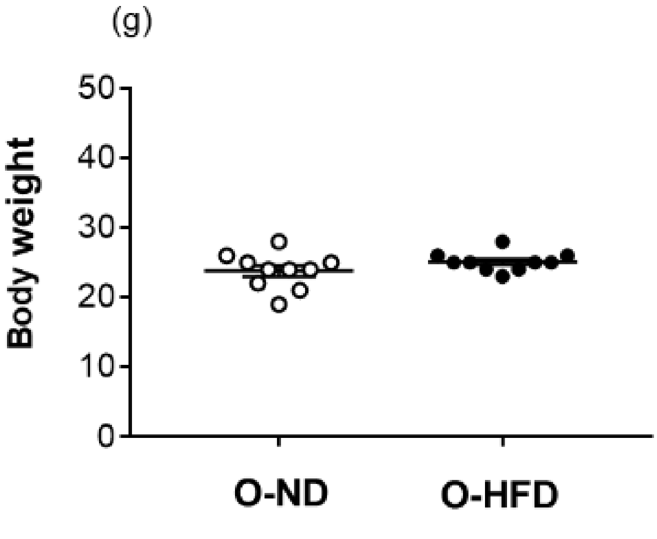

Fig. S2. Body weight of offspring. Values are mean ± SE for 10 O-ND and 10 O-HFD at 8 weeks of age. **p* < 0.05 vs. O-ND. Statistical analysis made by Student t test. O-ND, offspring of ND-fed dam; O-HFD, offspring of HFD-fed dam.

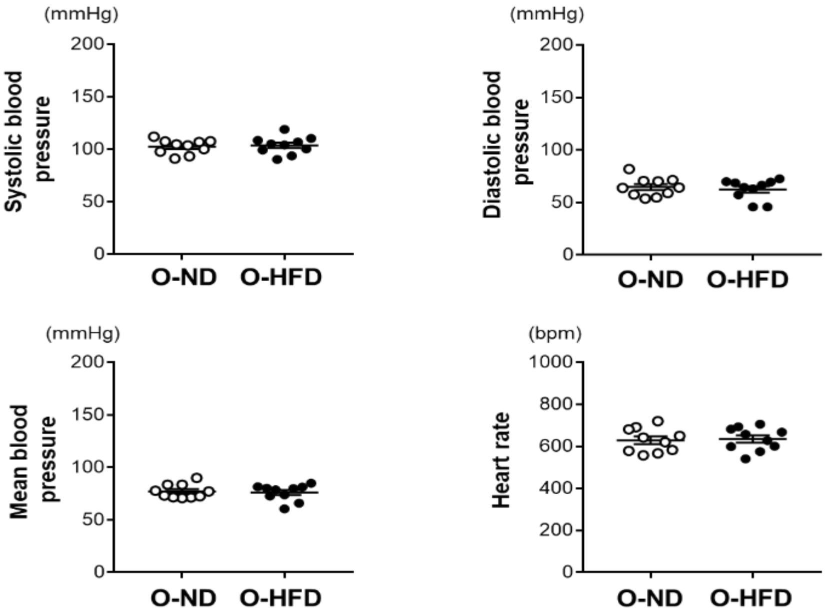

Fig. S3. Blood pressure and heart rate are comparable between O-ND and O-HFD. Values are mean ± SE for 10 O-ND and 10 O-HFD mice. Statistical analysis made by Student t test except for diastolic blood pressure (Mann–Whitney U test). O-ND, offspring of ND-fed dam; O-HFD, offspring of HFD-fed dam.

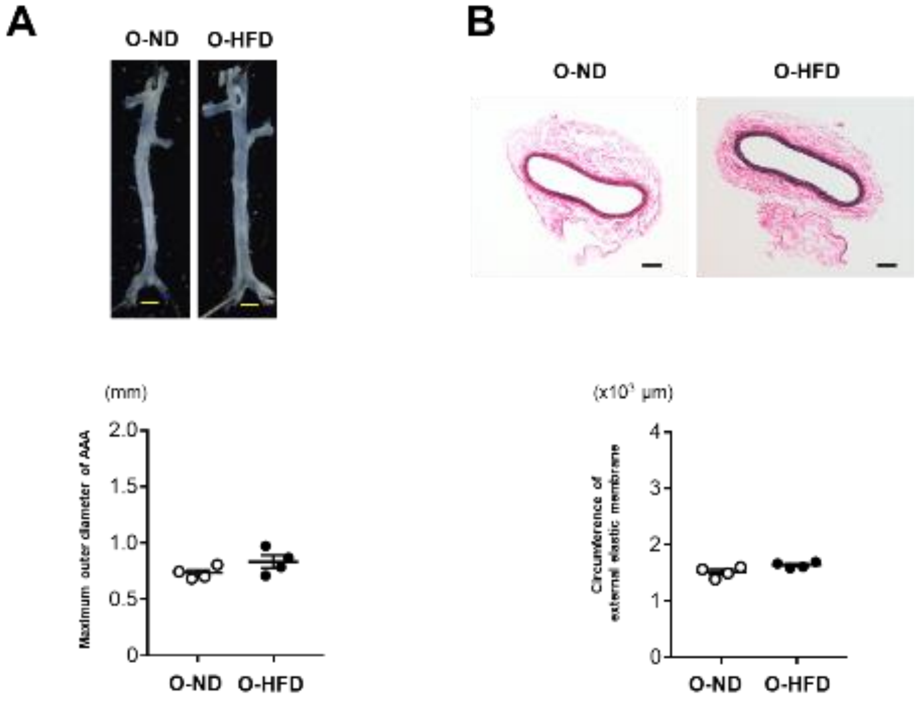

Fig. S4. Maternal HFD does not affect AAA development in sham-operated offspring. (A) Representative photographs and quantitative measurements of maximum outer diameters at 8 weeks after saline application. Values represent mean ± SEM for 4 O-ND and 4 O-HFD mice. Statistical analysis made by Student t test. O-ND, offspring of ND-fed dam; O-HFD, offspring of HFD-fed dam. Scale bar = 1 mm. (B) Representative photographs and quantitative measurements of circumferences of external elastic membranes before and at 8 weeks after saline application. Values represent mean ± SEM for 4 O-ND and 4 O-HFD mice. Statistical analysis made by Student t test. O-ND, offspring of ND-fed dam; O-HFD, offspring of HFD-fed dam. Scale bar = 100 μm.

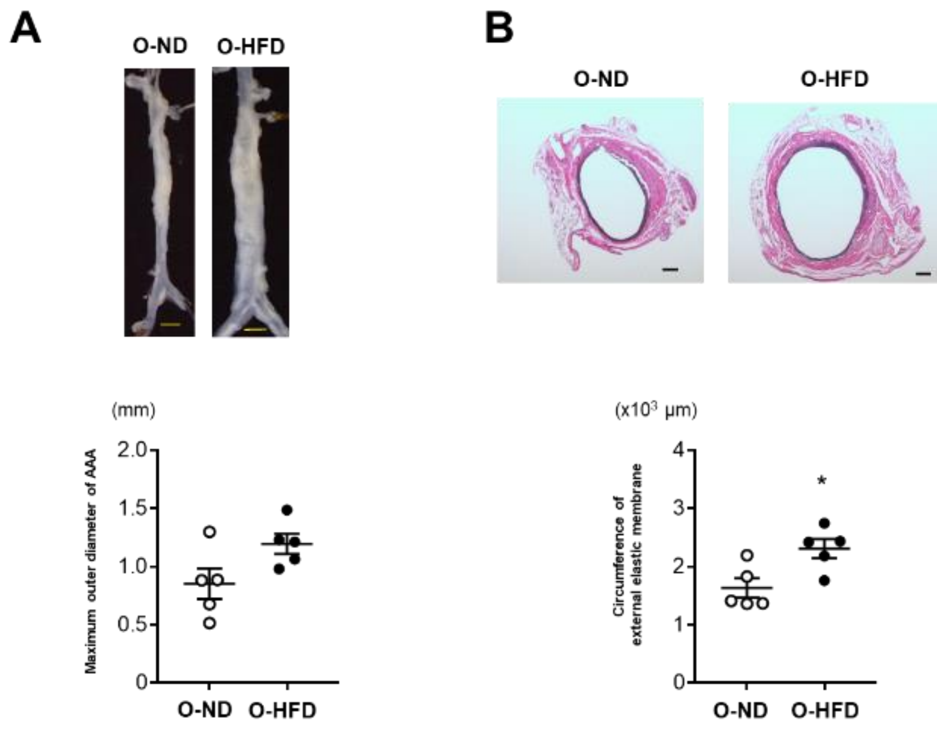

Fig. S5. Maternal HFD exaggerates the development of AAA in female offspring. (A) Representative photographs and quantitative measurements of maximum outer diameters at 4 weeks after $CaCl_2$ application. Values represent mean ± SEM for 5 O-ND and 5 O-HFD mice. Statistical analysis made by Student t test. O-ND, offspring of ND-fed dam; O-HFD, offspring of HFD-fed dam. Scale bar = 1 mm. (B) Representative photographs and quantitative measurements of circumferences of external elastic membranes at 4 weeks after $CaCl_2$ application. Values represent mean ± SEM for 5 O-ND and 5 O-HFD mice. *$p < 0.05$ vs. O-ND; Statistical analysis made by Student t test. O-ND, offspring of ND-fed dam; O-HFD, offspring of HFD-fed dam. Scale bar = 100 µm.

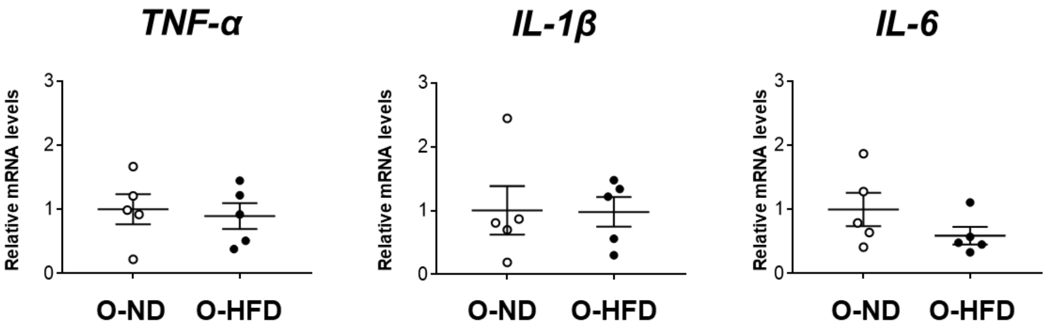

Fig. S6. Maternal HFD does not affect mRNA expression levels of proinflammatory cytokines. Quantitative PCR analysis of mRNA expression levels of TNF-α, IL-1β, and IL-6 in AAA at 1 week after CaCl2 application. Values represent mean ± SEM relative to O-ND. Each group consisted of 5 O-ND and 5 O-HFD mice. Statistical analysis made by Student t test. O-ND, offspring of ND-fed dam; O-HFD, offspring of HFD-fed dam. TNF-α, tumour necrosis factor-α; IL-1β, interleukin-1β; IL-6, interleukin-6.

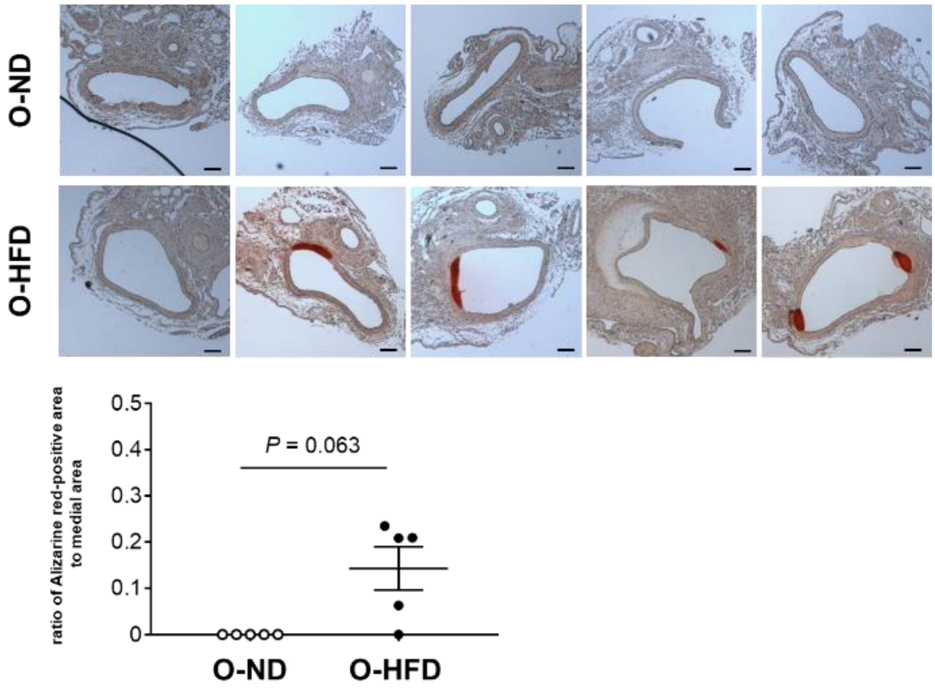

Fig. S7. Alizarine red staining in AAA. Representative photographs and quantitative measurements of the ratio of Alizarine red-positive area to medial area at 1 week after $CaCl_2$ application. Values represent mean ± SEM for 5 O-ND and 5 O-HFD mice. Statistical analysis was performed by Mann–Whitney U test. O-ND, offspring of ND-fed dam; O-HFD, offspring of HFD-fed dam. Scale bar = 100 μm.

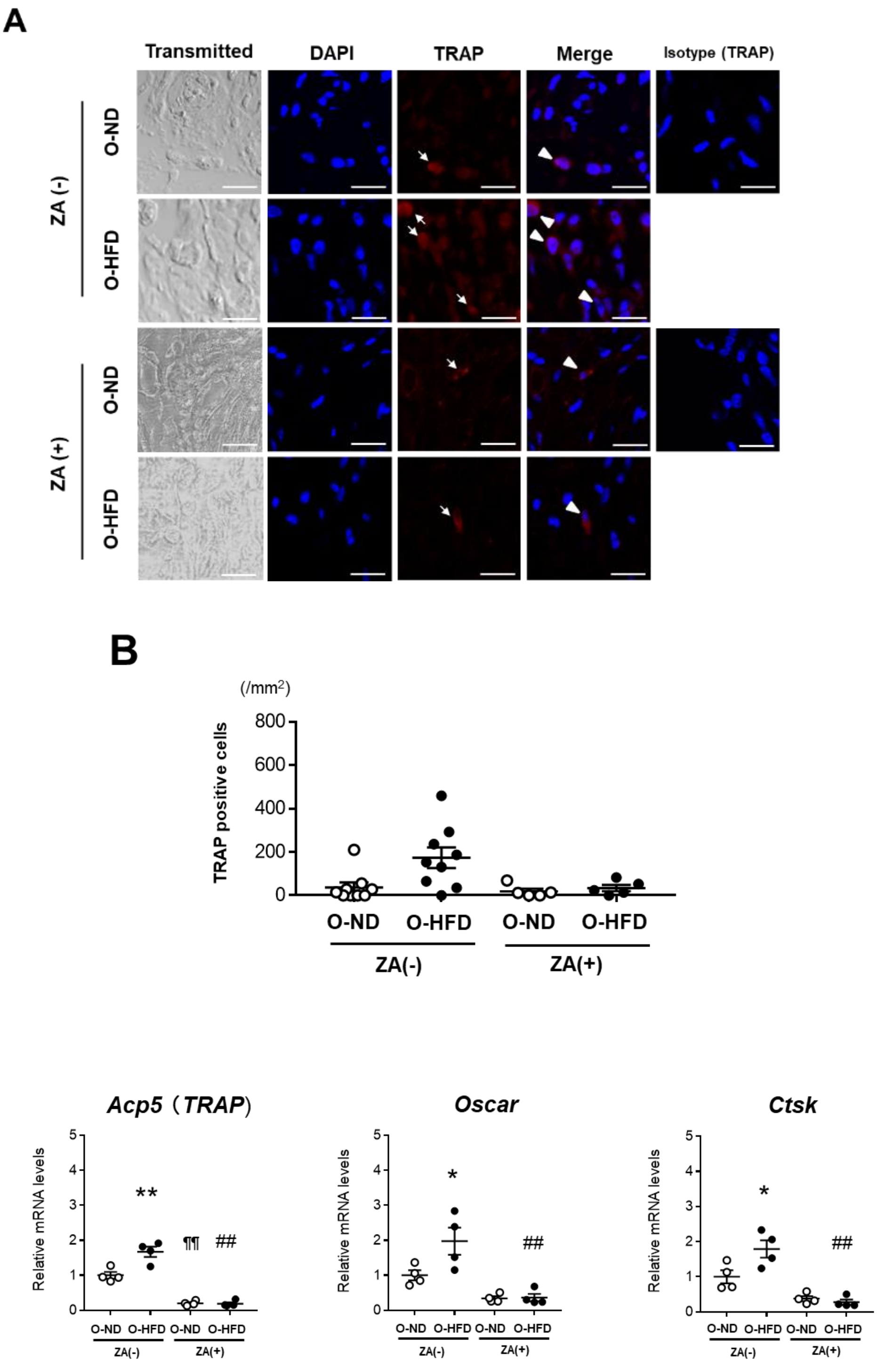

Fig. S8. Effect of ZA treatment on TRAP-positive cells and osteoclast-specific genes expression. **(A and B)** Representative photographs and quantitative measurements of the number of TRAP-positive cells at 1 week after $CaCl_2$ application. Values represent mean ± SEM for 5 O-ND and 5 O-HFD mice. Statistical analysis made by Kruskal-Wallis test. O-ND, offspring of ND-fed dam; O-HFD, offspring of HFD-fed dam. Scale bar = 25 µm. **(C)** Quantitative PCR analysis of mRNA expression levels of osteoclast differentiation-related genes. Values represent mean ± SEM relative to O-ND. Each group consisted of 4 O-ND and 4 O-HFD BMDMs. $^{*}p < 0.05$ vs. O-ND without ZA treatment. $^{\#}p < 0.05$ vs. O-HFD without ZA treatment; one-way ANOVA with Tukey-Kramer post hoc test. ZA, zoledronic acid; Acp5, tartrate-resistant acid

phosphatase type 5; Ctsk, cathepsin K. O-ND, offspring of ND-fed dam; O-HFD, offspring of HFD-fed dam.

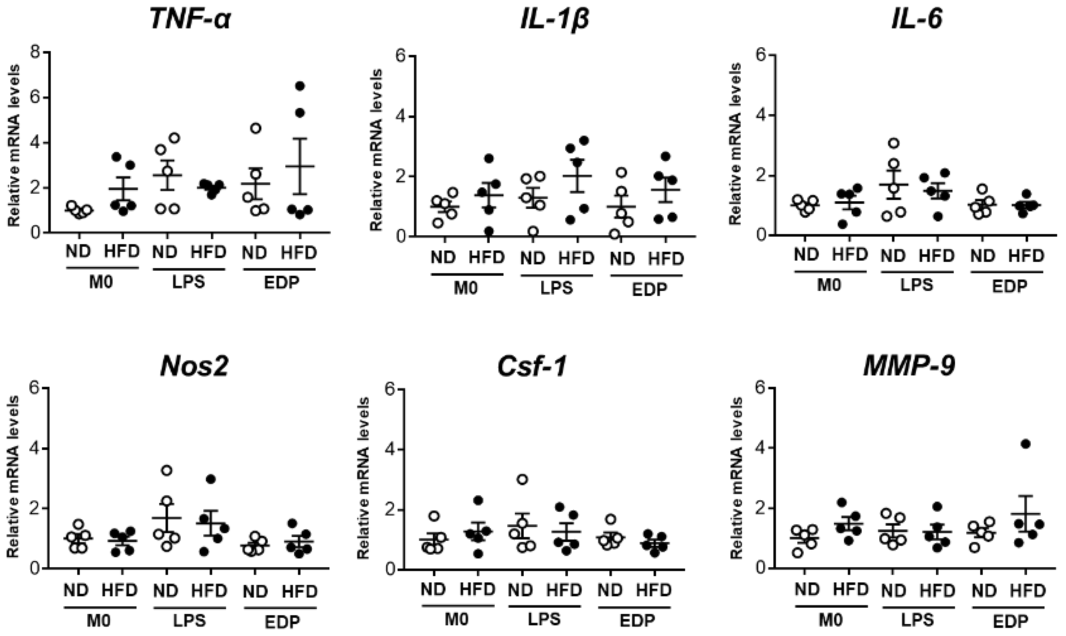

Fig. S9. Maternal HFD does not affect BMDM polarization into classically activated macrophages (M1). Quantitative PCR analysis of mRNA expression levels of M1 macrophage-related genes after stimulation with LPS or EDP. Values represent mean ± SEM relative to O-ND. Each group consisted of 5 O-ND and 5 O-HFD BMDMs. Statistical analysis made by Kruskal-Wallis test. O-ND, offspring of ND-fed dam; O-HFD, offspring of HFD-fed dam; LPS, lipopolysaccharide; EDP, elastin derived peptides; TNF-α, tumour necrosis factor-α; IL-1β, interleukin-1β; IL-6, interleukin-6; NOS2, nitric oxide synthase 2; Csf-1, colony stimulating factor 1; MMP-9, matrix metalloproteinase-9.
